# Gingival fibroblasts behavior on bioactive zirconia and titanium dental implant surfaces produced by a functionally graded technique

**DOI:** 10.1590/1678-7757-2020-0100

**Published:** 2020-07-13

**Authors:** Mariana Brito da CRUZ, Joana Faria MARQUES, Beatriz Ferreira FERNANDES, Mafalda COSTA, Georgina MIRANDA, António Duarte Sola Pereira da MATA, João Manuel Mendez CARAMES, Filipe Samuel SILVA

**Affiliations:** 1 Universidade de Lisboa Faculdade de Medicina Dentária LIBPhys Lisboa Portugal Universidade de Lisboa, Faculdade de Medicina Dentária, Grupo de Investigação em Biologia e Bioquímica Oral, LIBPhys, Lisboa, Portugal.; 2 Universidade do Minho Centro para Sistemas Micro Eletromecânicos Departamento de Engenharia Mecânica Guimarães Portugal Universidade do Minho, Centro para Sistemas Micro Eletromecânicos (CMEMS), Departamento de Engenharia Mecânica, Guimarães, Portugal.; 3 Universidade de Lisboa Faculdade de Medicina Dentária Bone Phisiology Lab Lisboa Portugal Universidade de Lisboa, Faculdade de Medicina Dentária, Bone Phisiology Lab, LIBPhys, Lisboa, Portugal.

**Keywords:** Titanium, Zirconium oxide, Dental implants, Fibroblasts, Functionally graded materials

## Abstract

Adding a biological apatite layer to the implant surface enhances bone healing around the implant.

**Objective:**

This study aimed to characterize the mechanical properties and test human gingival fibroblasts behavior in contact with Zirconia and Titanium bioactive-modified implant materials.

**Methodology:**

6 groups were considered: Titanium (Ti6Al4V), Ti6Al4V with 5% HA and 5% ßTCP, Zirconia (YTZP), YTZP with 5% HA and 5% ßTCP. For each group, we produced discs using a novel fabrication method for functionally graded materials, under adequate conditions for etching and grit-blasting to achieve equivalent surface microroughness among the samples. Surface roughness (Ra, Rz), water contact angle, shear bond strength, and Vickers hardness were performed. Human gingival fibroblasts immortalized by hTERT gene from the fourth passage, were seeded on discs for 14 days. Cell viability and proliferation were assessed using a resazurin-based method, and cellular adhesion and morphology using field emission gun scanning electron microscopy (FEG-SEM). After 3 days of culture, images of fluorescent nucleic acid stain were collected by confocal laser scanning microscopy (CLSM).

**Results:**

Results were presented as mean ± standard deviation (SD). We compared groups using one-way ANOVA with Tukey’s post-hoc test, and significance level was set at p<0.05. After 14 days of culture, cell viability and proliferation were significantly higher in YTZP group than in other groups (p<0.05). Samples of YTZP-ßTCP presented significantly higher wettability (p<0.05); yet, we observed no improvement in cell behavior on this group. Fibroblast spreading and surface density were more evident on YTZP specimens. Adding calcium-phosphate bioactive did not alter the tested mechanical properties; however, Ti6Al4V material shear bond strength was statistically higher than other groups (p<0.05).

**Conclusion:**

Adding bioactive materials did not improve soft-tissue cell behavior. When compared to other zirconia and titanium groups, pure zirconia surface improved adhesion, viability and proliferation of fibroblasts. Cell behavior seems to depend on surface chemical composition rather than on surface roughness.

## Introduction

Titanium or titanium alloys (Ti6Al4V) presents excellent biocompatibility and mechanical properties, being the material of choice for producing dental implants.^1,2^ Yet, metal-free restorations provide a viable option to meet the increasingly higher aesthetic standards.^3,4^ Yttria-stabilized Zirconia (YTZP) was introduced as an alternative for titanium implants due to its favorable biological, mechanical, and aesthetic properties.^5^Clinical evidence suggests that these two materials exhibit comparable osseointegration behavior,^6,9^ and soft-tissue favorable response to zirconia implants is widely reported.^10,11^Osseointegration is the core of a successful endosseous oral implant, depending on the chemical, physical, mechanical, and topographic characteristics of the surface.^1,12^

Zirconia surface have been modified to increase roughness and bioactivity, improving function and cellular responses.^13,14^ According to the literature, adding a biological apatite layer enhances bone healing around the implant.^1^For years, hydroxyapatite (HA) or beta tricalcium phosphates (βTCP) have been used, achieving promising outcomes.^15,16^ Various methods have been employed into coating metal implants,^1^within which one of the major concerns is possibly delaminating the surface of the titanium implant and failing at the implant-coating interface.^17^Considering that, we developed a fabrication method, which combines Functionally Graded Materials (FGM) with hot pressing, to produce composite materials with the advantages of the biological bioactive calcium phosphate layer, and without the potential risk of delamination.^18,19^ The FGM technique comprises the formation of gradients of chemical composition, and phases distribution or microstructure.^20^ The idea was creating an implant which outer layer had a percentage of bioactive compounds integrated into the implant matrix, whereas the inner layer was composed solely by YTZP or Titanium; this would guarantee both the mechanical properties within the implant core, and the bioactive properties (in contact with the surrounding tissue) in its outside area. Titanium and zirconia materials – modified with these bioactive compounds, and using this strategy – have already been observed to enhance osteoblast activity.^21,22^ Osteoblastic cell behavior has been widely characterized for bioactive calcium phosphate materials, but not much is known regarding cellular events involving soft tissue response and fibroblast adhesion.

This study aimed to characterize the mechanical features, and evaluate and compare human gingival fibroblasts behavior in contact with zirconia and titanium bioactive-modified implant materials produced with equivalent surface roughness.

## Methodology

### Substrates

As previously described, we have used hot pressing technique to produce functionally graded materials.^21,23^ Ti6Al4V powders were mechanically mixed with hydroxyapatite (HA) or beta-tricalcium phospate (ßTCP) in a proportion of 95 wt % Ti6Al4V, and 5 wt % HA or ßTCP, using a stainless steel jar containing steel mill balls, at 25 rpm for 21 hours. The powder mixture was dehydrated at 110ºC for 1 h and placed into graphite molds. The mold was placed inside a primary vacuum chamber and hot-pressed, producing the discs. Samples were then compressed at 2 bar and heated up to 1200ºC at 31ºC/min. At 1100ºC, the pressure was raised at 20 MPa and maintained during 30 min.^21^

YTZP samples with HA or βTCP were prepared with separated powders, immersed in ethanol and dispersed under a high energy ultrasonication process (40KHz, 200W) for 30s.^22^ Then, YTZP granules were added to the solution while each bioactive material was in suspension for a homogeneous mixing. To avoid decantation, the alcohol volume in the solutions was controlled; then, to evaporate the ethanol, they were heated on a furnace at 60ºC for 1h and 30 min. YTZP powders were mechanically mixed in a proportion at 95%(v/v) YTZP and 5%(v/v) HA or βTCP, or maintained pure, using a stainless steel jar containing steel mill balls, at 25 rpm for 21 h. Powders were placed in parallel into a pressing mold, separated by a thin plastic frame that was later removed, pressed at 200 MPa under uniaxial pressing, and sintered at a heating and cooling rate of 8 °C/min up to 1500°C (Zirkonofen 700 furnace, Zirkonzhan, Italy) for 2 h. (22) Table 1 lists YTZP powder chemical composition.

**Table 1:** Chemical composition of 3Y-TZP powder (according to manufacturer Tosoh) (Tosoh Corporation©, Amsterdam, Netherlands)

Element	Y2O3	Al2O3	SIO2	Fe2O3	Na2O	ZrO2 + HfO2 + Y2O3 + Al2O3
Wt %	5.15 ± 0.20	0.25 ± 0.10	≤ 0.02	≤ 0.01	≤ 0.04	> 99.9

In total, 48 discs with 8mm of diameter and 3 mm of height were produced and divided in six groups (N=8): Ti6Al4V, Ti6Al4V containing 5% HA (Ti6Al4V-HA) , Ti6Al4V containing 5% ßTCP (Ti6Al4V-ßTCP), YTZP , YTZP containing 5% HA (YTZP-HA) or YTZP containing 5% βTCP (YTZP-βTCP). Specimens were wet ground with SiC abrasive papers down to 4000 mesh, polished till a mirror-like finishing using aluminum oxide suspension (1 µm), and then cleaned ultrasonically. Surfaces were etched with 5% HNO_3_, 10% HF and 85% distilled water. Discs were then air-braded using 100 µm alumina particles, under appropriate conditions for obtaining similar roughness for all samples. Finally, samples were ultrasonically cleaned in 100% ethanol for 10 min, and autoclave-sterilized at 121ºC for 20 min.24

### Mechanical characterization

Before mechanical and biological tests, one sample within each group was inspected under Ultra-high-resolution Field Emission Gun Scanning Electron Microscopy (FEG-SEM) - FEI NOVA 200 Nano SEM, FEI, Oregon, USA, which obtained micrographs at 500x magnification, with 10 kV acceleration voltage. Backscattering Electron (BSED), images were acquired at an acceleration voltage of 15 kV.^24^

Shear tests were carried out with composite samples to measure the maximum stress that the material was able to support before rupturing: samples were positioned half fixed in a metallic support and half exposed – portion in which the cutting insert acted. This test was conducted in a servo hydraulic machine (Instron 8874) with a 25 kN capacity load cell and a 0.02 mm/s crosshead speed at room temperature. The maximum shear stress was determined by the ratio between maximum load and cross-section area (n=3).^22^ For the vickers hardness tests, a Vickers micro-hardness tester (DuraScan, emcotest, Germany) was used on 4.9N (500g) loading for 15s; the average hardness was calculated from five indentations on each of three different groups.^21,22^ Roughness was measured by determining Ra and Rz values, according to ISO 4287-1997, using a contact profilometer (Surftest SJ 201 from Mitutoyo, Japan), at a 4 mm evaluation length, a 0.8 mm cut-off wavelength, and a 0.25 mm/s scan speed (n=3). Roughness average (Ra) was determined as average length between peaks and valleys and deviation from mean line, and the peak-to-valley roughness (Rz) as average vertical distance between the highest and the lowest peak.

Wettability was assessed by contact angle evaluation, using the drop shape analysis: a water droplet was deposited on the surface and, after stabilizing it, we determined four different measurements for each group by image processing using a digital goniometer (OCA 20, Data Physics, Germany).^22^

### Biological characterization

#### Fibroblast culture

Immortalized Human Gingival Fibroblasts (HGF; Applied Biological Materials Inc., Richmond, BC, Canada) were obtained from primary cells detached from a gingival biopsy of normal tissue, conditionally immortalized by hTERT gene. Cells grew in 75 cm^2^ flasks (Corning, Corning NY, USA), on a 5% CO_2_,100% humidity, and at 37ºC environment. The culture medium was a Dulbecco’s Modified Eagle’s Medium – DMEM (Biowhittaker, Lonza^TM^, Walksville, USA) supplemented with 10% fetal bovine serum (Biowest, Nuaillé, France). After reaching 80% confluence, cells were detached using trypsin-EDTA (Lonza, Veners, Belgium), centrifuged at 800 rpm, and resuspended in culture medium. Cells were seeded at a density of 1 x 10^4^ cells/well in 0.5mL medium. The medium was changed every 48h and all tests were performed on the fourth subculture.^22,24^

#### Cell Viability and proliferation Assay of Fibroblasts

Six groups were analyzed: Ti6Al4V, Ti6Al4V-HA, Ti6Al4V-ßTCP, YTZP, YTZP-HA and YTZP-βTCP. Sample discs (N=8) were placed in 48-well culture plates (Corning, Corning NY, USA) under sterile conditions and cell viability and proliferation were measured after 1, 3, 7 and 14 days using a resazurin-based viability assay - Cell-Titer Blue^®^, (Promega, Madison, WI, USA) according to the manufacturer’s protocol. Fluorescence spectroscopy (PerkinElmer LS 50B, Waltham MA, USA) at excitation/emission wavelengths of 560/590 nm was performed and results were presented in fluorescence arbitrary units (AU).^22,24^

#### Cell morphology of fibroblasts

After 1 and 7 days of culture (5% CO_2_, 37 ºC), samples were washed with phosphate buffer saline (PBS) and fixed in 1.5% (v/v) glutaraldehyde solution for 10 min. Then, a dehydration process was performed by serial dilutions in ethanol. Samples were coated with a 15 nm thin film composed of Au-Pd (80-20 weight %) using a high-resolution sputtering coater (208HR Cressington Company, Watford, United Kingdom), coupled to a MTM-20 Cressington High Resolution Thickness Controller (Cressington Company, Watford, United Kingdom). Morphological analyses were performed by FEG-SEM (FEI NOVA 200 Nano SEM, FEI, Oregon, USA). Samples were inspected with secondary electrons mode at 10kV, and at different magnifications (500X and 1000X). Atomic contrast images were acquired by Backscattering Electron Detector (BSED) mode at 15 kV. Two calibrated researchers analyzed the images, focusing on cell morphology, spreading, and early contact with materials.^24^

#### Fluorescent staining of Nucleic Acids

At 3 days of culture, discs cultured with fibroblasts were removed and cells were fixed in 1.5% glutaraldehyde solution, and DAPI-stained them (Sigma-Aldrich D9542, Hampshire, UK). Nucleic acid stained images were obtained by spectrophotometry at 460 nm wavelength, using a Leica TCS SP٥ confocal microscope (Leica Microsystems, USA) coupled to v2.0 LAS AF LITE software (Leica Microsystems, USA).^24^

## Statistical analysis

Shapiro-Wilk test was used for checking the normal distribution of data. For determining significant differences among groups for mechanical and biological tests, a factorial analysis of variance (one-way ANOVA) or Mann-Whitney tests were used when appropriate. Tukey’s post-hoc test was applied to identify significant differences among groups, considering *p* < 0.05 as significance level. Data is presented as mean ± standard deviation (SD). All statistical analyses were performed using IBM® SPSS® 24.0 statistics software for Mac (SPSS, Chicago, USA).

## Results

### Mechanical Properties

**Figure 1:**
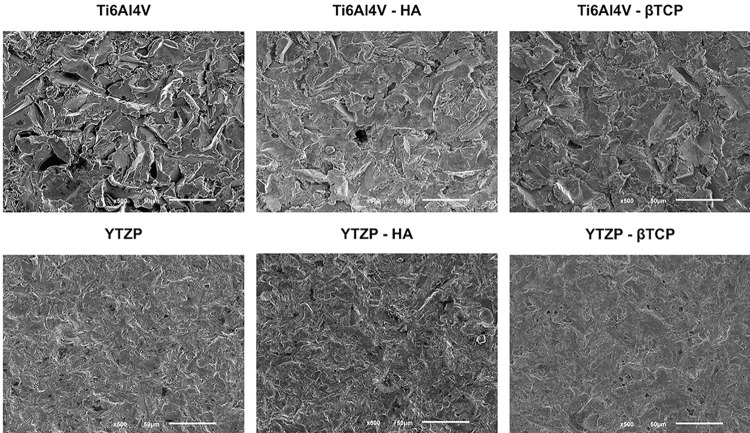
FEG-SEM micrographs after surface treatment of Ti6Al4V, Ti6Al4V-HA, Ti6Al4V-ßTCP, YTZP, YTZP-HA, YTZP-ßTCP samples (150x magnification)

As Figure 1 shows, before mechanical and biological tests, we observed all specimens using FEG-SEM. Preliminary micrographs confirmed similar surface roughness in all study groups, resulting from surface treatment.

**Figure 2:**
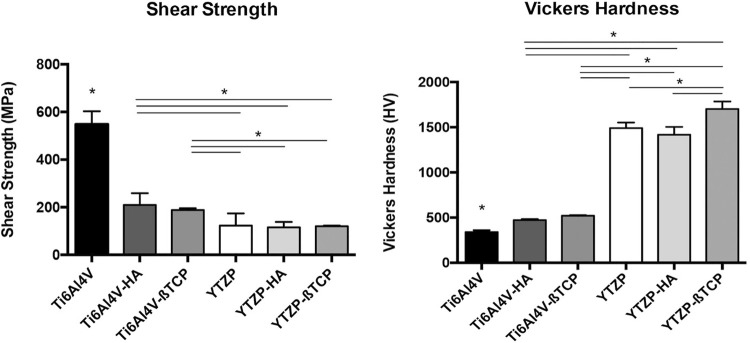
(A) Shear strength and (B) Vicker’s hardness values recorded for Ti6Al4V, Ti6Al4V-HA, Ti6Al4V-ßTCP, YTZP, YTZP-HA, and YTZP-ßTCP materials as mean and standard deviation

Figure 2 shows shear bond strength and Vickers micro-hardness values . Shear bond strength test (Figure 2A) showed that Ti6Al4V group had the highest mean value (*p*<0.05). Although no statistically significant differences were found between Ti6Al4V-HA and Ti6Al4V-ßTCP, both showed higher values than all YTZP groups. Adding bioactive calcium-phosphate compounds to YTZP groups entailed no statistically significant differences when compared to pure YTZP implant materials (p > 0.05). Vickers hardness (Figure 2B) results showed lower values for Ti6Al4V than for all other groups (*p*<0.05); Ti6Al4V-HA and Ti6Al4V-ßTCP showed lower values than YTZP groups (*p*<0.05), but no statistically significant differences were observed among them. Regarding pairwise comparisons among YTZP-based groups, YTZP-ßTCP presented the highest micro-hardness values (*p*<0.05).

Surface measurement and hydrophilicity determination characterized all groups’ surfaces. Table 2 presents samples roughness values of Ra (µm) and Rz (µm), as well as surface hydrophilicity by water contact angle (º). The results showed similar Ra values for all groups, with no statistically significant differences (*p*>0.05); but YTZP presented the lowest Rz values (*p*<0.05).

**Table 2:** Contact angle (º) and Roughness values – Ra (µm) and Rz (µm) for Ti6Al4V, Ti6Al4V-HA, Ti6Al4V-ßTCP, YTZP, YTZP-HA, YTZP-ßTCP surfaces as mean and standard deviation

	Roughness values - R_a_	Standard deviation	Roughness values - R_z_	Standard deviation	Contact angle (º)	Standard deviation (º)
	(μm)	(μm)	(μm)	(μm)		
Ti6Al4V	2.05	0.120	14.57	1.2	89.66	1.52
Ti6Al4V-HA	2.08	0.11	13.89	0.71	81,74	1.89
Ti6Al4V-ßTCP	2.19	0.08	14.93	1.61	75.29	1.23
YTZP	1,45	0,11	8.92*	0.91	70.59	1.73
YTZP-HA	1.76	0.07	11.26	0.19	70.82	2.45
YTZP-ßTCP	1.86	0.10	12.31	1.19	65.04	1.92

Regarding water contact angle, the results were statistically different among all groups in pairwise comparisons (*p* <0.05), except between YTZP and YTZP-HA (*p*>0.05).

#### Cell viability and proliferation

**Figure 3:**
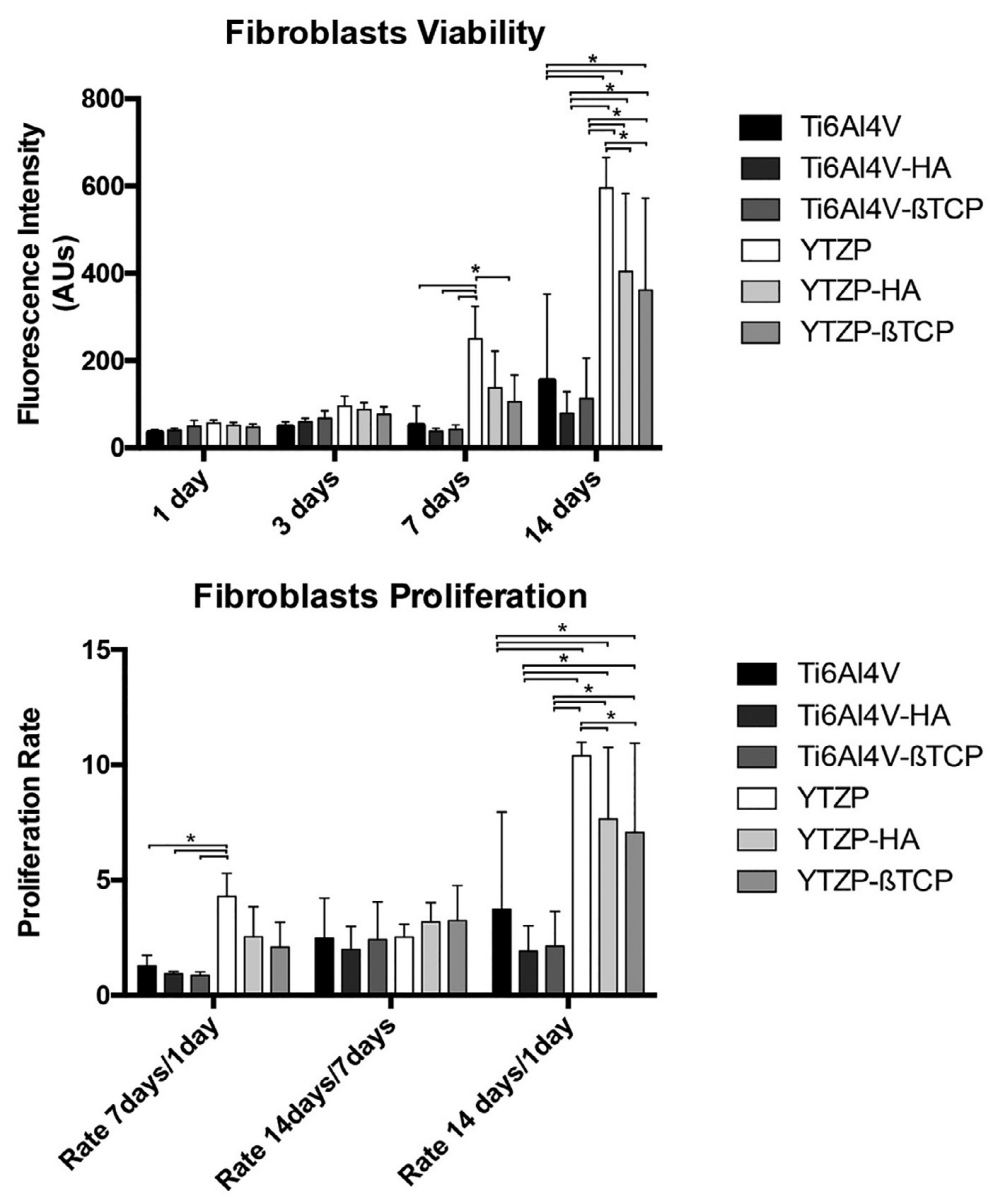
Bar charts showing the fibroblast viability of Ti6Al4V, Ti6Al4V-HA, Ti6Al4V-ßTCP, YTZP, YTZP-HA, and YTZP-ßTCP as mean, using fluorescence intensity expressed in arbitrary units; and fibroblast proliferation rates as mean calculated by the ratio of fluorescence intensity at 7 days/ 1 day, 14 days/7 days and 14 days / 1day. Error bars represent standard deviation. For comparing study groups, repeated measures one-way ANOVA with post-hoc Tukey test was used. Statistical significance: *p < 0.05

Figure 3 shows the results of cell viability and proliferation after 1, 3 ,7 and 14 days. Until 7 days, fibroblast culture showed similar viability among groups, without statistically significant differences. At 7 days, YTZP presented the highest viability among all groups (*p*<0.05), except for YTZP-HA. At 14 days, YTZP presented the highest viability among all groups (*p*<0.05); likewise, YTZP-HA and YTZP-ßTCP presented higher viability than all Ti6Al4V groups (*P*<0.05).

YTZP group showed the highest fibroblast proliferation rate (*p*<0.05). After 14 days of culture, YTZP-HA and YTZP-ßTCP presented higher proliferation rates than Ti6Al4V, Ti6Al4V-HA and Ti6Al4V-ßTCP (*p*<0.05).

#### Cell morphology

**Figure 4:**
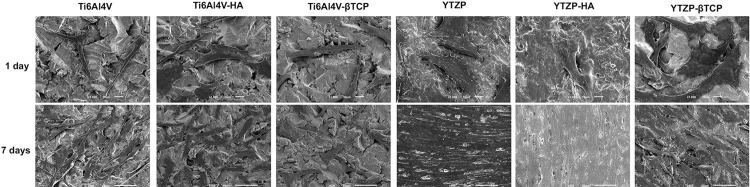
FEG-SEM micrographs of fibroblasts cultured on Ti6Al4V, Ti6Al4V-HA, Ti6Al4V-ßTCP, YTZP, YTZP-HA, and YTZP-ßTCP surfaces at 1 day with x1000 magnification, and 7 days with x500

Figure 4 portrays cell adhesion and fibroblasts morphology on test samples after 1 and 7 days with corresponding magnifications. After 1 day of culture, FEG-SEM micrographs showed adherent cells in all groups, with similar number of cells spread on each surface. However, we found different cells spreading between YTZP- and Ti6Al4V-based surfaces. At 7 days, the differences persisted – mostly related to the increase in the number and dissemination of cells along pure YTZP and YTZP-HA surfaces. Pure YTZP material showed higher cell density than all other groups.

**Figure 5:**
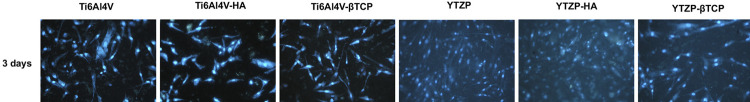
Fluorescence photomicrographs of DAPI – stained fibroblasts cultured for 3 days on Ti6Al4V, Ti6Al4V-HA, Ti6Al4V-ßTCP, YTZP, YTZP-HA, and YTZP-ßTCP specimens. Images are representative of 3 replicates

Figure 5 shows fluorescent staining of fibroblast nucleic acids cultured on discs. We observed that, at 3 days in culture, YZTP group presented a higher number of fibroblasts than all other materials, in particular Ti6Al4V, Ti6Al4V-HA and Ti6Al4V-BTCP. These observations confirm the previously described results of initial adhesion, as observed by FEG-SEM.

## Discussion

This study is pioneer in characterizing soft-tissue cell behavior in contact with a new FGM-based dental implant design that uses either Zirconia or Titanium substrates embedded with bioactive calcium-phosphate particles. The implant component in contact with gingival connective tissue cells (abutment) is often smooth; this study aimed to compare fibroblast and osteoblasts for elucidating surface chemistry role on the differential behavior of these cells. This model enabled the simulation of the connective tissue cells response when in contact with implant surface – as occurs with bone loss or peri-implantitis events, when a new epithelial attachment is formed. Most studies on osteoblasts have used surfaces roughness around 1 and 2 µm, so we produced surfaces within these same values. We assessed the mechanical features of these materials to provide a detailed characterization. Previous studies assessing different materials for implant surfaces are often biased regarding surface roughness, which prevents them to discriminate the individual contributions of the chemical composition from the material biological behavior.^5,25,26^ Our study adopted a thorough methodology to produce equivalent surface roughness among all samples, and comparable to most surfaces already in the market, so that its chemical composition and related properties could be independently evaluated.

### Mechanical properties

Previous studies have incorporated bioactive materials into implant materials by the FGM technique , obtaining promising outcomes on mechanical behavior. However, scientific support for its biological behavior in contact with FGM samples are still insufficient.^21^Our study hypothesizes that by incorporating bioactive particles, the shear strength of the composite materials would decreased. Yet, micro-hardness results have suggested that adding calcium-phosphate compounds increase materials microhardness. This is explained because, adding these hard bioactive materials as a reinforcement to increase composite overall microhardness causes shear values to decrease as (despite their increased hardness) these materials brittleness would act as fragile areas and reduce the effective resistance of the cross-section subjected to shear. We did not observe it for YTZP-based surfaces, which confirm other author’s previous reports.^15^ Regarding Ti6Al4V bioactive composite, the bioactive materials (HAp and bTCP) acted as fragile areas, compromising the metal ductile natural behavior. Conversely, YTZP composite comprises a ceramic matrix which is already a fragile material; then, adding the bioactive materials apparently did not affect this composite shear behavior. These results may be related to binomial ceramic-ceramic better chemical affinity and thermal compatibility (YTZP – HAp/bTCP) than metal-ceramic (Ti6Al4V – HAp/bTCP). Considering that this composite is only present in the implant outer layer, despite shear strength decrease, the overall mechanical properties are ensured by the implant internal layer. Regardless of these encouraging preliminary results, additional tests and a more comprehensive mechanical characterization are required to fully validate this FGM strategy.

### Roughness and contact angle

Several experimental investigations have illustrated the influence of implant surface topography on bone responses.^27,28^ Some studies postulate that the surface roughness of titanium implants is the key parameter for osseointegration rate and quality.^29^ However, the exact role of chemistry and topographical parameters of implants surfaces in the early events of bone integration is still unclear.^1^ The greatest doubts are which surface parameters are paramount to osseointegration, and whether they are as important to fibroblast behavior. Considering that, we have adopted the uniform Ra values of 1–2 μm, for being widely applied in implant dentistry and largely reported for improved osseointegration and clinical outcomes.^28^ Although samples achieved equivalent values of Ra, we observed slight differences regarding Rz values among our study groups. Rz averages are calculated by the five highest peaks and the five deepest valleys.^3^ The different method and results between Rz and Ra may be explained for the exceptionally high peaks or low valleys in sample surfaces, instead of uniform variations in height. Ra averages all measurements within a given sample; thus, extreme points are blended into the average, and the method is unable to discriminate them.^16,30^

Surface hydrophilicity is also an important feature associated with cell response. Strong hydrophilic surfaces are deemed more appropriate for a favorable biological response, considering their enhanced affinity with biological fluids, cells, and surrounding tissues. In titanium implant surfaces, contact angle measures ranged from 0º (hydrophilic) to 140º (hydrophobic).^28,31^ However, our results showed that hydrophilicity does not affect fibroblast behavior in the same way as it does for osteoblasts: cell adhesion, viability, and proliferation were higher on pure YTZP, although YTZP-ßTCP was the most hydrophilic material. Even though, YTZP-based groups showed a higher hydrophilicity and improved cell behavior than titanium samples. We found that surface hydrophilicity may be an important characteristic for implants, but chemical composition seems more important in fibroblast cell modulation. As in other studies, we encountered some difficulties in isolating surface variables for independently studying their effects, as most of these parameters are related to cell modulation.^32-34^

### Cell behavior

This study deployed an Immortalized cell line of Human Gingival Fibroblast, with similar morphology and responses (compared to primary human gingival fibroblasts). A previous study conducted by our group revealed that these novel functionally-graded composites, based on Ti6Al4V and Hydroxyapatite or b-TCP embedded with osteoblasts, presented a bioactivity improvement when compared to pure Ti6Al4V.^21^ However, this study reported that pure YTZP materials presented improved fibroblast cell behavior on adhesion, viability, and proliferation than other groups did. This finding suggests that adding bioactive components as HA and b-TCP on implant surfaces did not improve gingival fibroblast behavior.

As suggested by other studies,^5,35,36^ YTZP materials showed an improved fibroblast behavior when comparing Titanium- and Zirconia-based surfaces (although equivalent cell responses have been reported in these).^5,35-38^ Some of these studies have used osteoblasts, and most of them did not standardize features such as surface roughness.^5,39^ According to the reviewed literature, one of ceramic implants key features is its reduced bacterial biofilm accumulation, improving soft-tissue management.^40,41^ Considering these antimicrobial properties, we tested Zirconia as a base material, and modified it with bioactive particles to enhance the cell responses of soft and hard biological tissues. However, as Zirconia alone produced optimal fibroblast responses, bioactive would not increase an already optimal soft tissue seal; thus, pure Zirconia should be preferred for ceramic implants in cervical regions.

This is an *in vitro* study; yet, it highlights the need for an implant design that present optimal physiochemical characteristics for specific cellular behavior in two different areas: osseointegration and gingival integration of dental implant surface. FGM technique enables other substances incorporation for improving cell behavior or controlling bacterial adhesion, besides providing an interesting perspective for developing materials able to withstand the most common risk factors threatening the long-term maintenance of oral implants.

This study has the inherent limitations of an *in vitro* study of cell behavior. Further studies should account for larger sample sizes and assess both differentiation and inflammatory markers. Differentiation markers offer a better understanding of cellular and molecular mechanisms, such as collagen type I and interleukin 8. However, data from *in vitro* studies are limited, so further *in vivo* studies with long follow-up should be considered for a precise knowledge regarding the relationship between these materials specific features and cell behavior in complex biological system.

## Conclusion

Adding bioactive ceramic materials by FGM technique has been performed with promising mechanical and cell behavior in osteoblasts. This study evaluated – through a comprehensive mechanical characterization – soft tissue-associated cells behavior in contact with FGM-based dental implant materials; these materials were developed using either Zirconia or Titanium substrates embedded with bioactive calcium-phosphate particles, and had equivalent roughness among the samples as well as already commercialized implants. Adding bioactive particles by FGM technique did not decrease the mechanical properties of zirconia-based materials. The results of this *in vitro* study suggest that Titanium and Zirconia bioactive-modified surfaces decreased adhesion, viability, and proliferation of fibroblasts when compared to pure materials, presenting optimal responses for pure YTZP.
